# Leveraging artificial intelligence community analytics and nanopore metagenomic surveillance to monitor early enteropathogen outbreaks

**DOI:** 10.3389/fpubh.2025.1675080

**Published:** 2025-11-26

**Authors:** Jeff Gauthier, Sima Mohammadi, Irena Kukavica-Ibrulj, Brian Boyle, Chrystal Landgraff, Lawrence Goodridge, Kenton White, Benjamin Chapman, Roger C. Levesque

**Affiliations:** 1 Département de Microbiologie-Infectiologie et d’Immunologie, Institut de Biologie Intégrative et des Systèmes, Université Laval, Quebec, QC, Canada; 2 National Microbiology Laboratory, Public Health Agency of Canada, Winnipeg, MB, Canada; 3 Food Science Department, University of Guelph, Guelph, ON, Canada; 4 Advanced Symbolics Inc., Ottawa, ON, Canada

**Keywords:** metagenomics, artificial intelligence, *Salmonella*, pathogen surveillance, Oxford nanopore sequencing

## Abstract

Foodborne enteric infections are a major public health and economical burden, yet their surveillance often relies on latent indicators that delay containment efforts by several days and weeks. Conversely, whole metagenome shotgun sequencing of communal wastewater allows continuous monitoring of enteric pathogens. Spikes in abundance can be observed several weeks before the first case reports emerge. In addition, AI-driven social media mining, already in use for public opinion analytics, could be repurposed for predicting outbreaks at the community level by predicting the number of people experiencing symptoms in the population given their social media activity. Here we report how AI-driven community analytics and high-throughput long-read metagenomic surveillance of communal wastewater microbiota were combined to monitor non-typhoidal salmonellosis in Quebec City, Canada, from August 2023 to February 2024. Both approaches indicated similar fluctuations over time for: (i) people experiencing salmonellosis symptoms, and (ii) *Salmonella enterica* relative abundance in wastewater, with predicted cases leading metagenomic peaks by a week. Moreover, both approaches detected a maximum around September 13th, 2023, 5 weeks before a *Salmonella* food recall for the Quebec and Ontario provinces was made by the Public Health Agency of Canada. We therefore suggest that continuous AI-driven analytics and wastewater metagenomics monitoring could become part of a nationwide surveillance pipeline from the community scale to the molecular level.

## Introduction

Enteric infections related to foodborne illnesses are a global public health burden, with estimates reaching ~1,5 million annual deaths and 1,020 disability-adjusted life-years per 100,000 population (1), while also costing billions of dollars in healthcare and food recalls (2). *Salmonella* spp. are estimated to account for causing roughly 155,000 deaths worldwide (3). Pathogen surveillance is therefore critical to prevent and control outbreaks. However, conventional monitoring methods often rely on latent indicators, such as case reports from hospitals and samples from infected individuals to both identify the causative agent and its prevalence. This process requires days, even weeks, before mitigating efforts can be deployed (2). Conversely, whole metagenome shotgun sequencing allows the sampling of all genetic material in a sample (4).

In parallel, several initiatives revealed the potential of artificial intelligence (AI) to enhance diagnostics, drug discovery and tailoring treatments based on personal genomics data (5). While useful at the patient level, the use of AI for public health (especially monitoring outbreaks) raises ethical questions. Notably, there are concerns regarding data privacy and the accuracy of predictions made by generative pre-trained transformer models (6). For these reasons, any AI-driven community data mining for predicting outbreaks should remain broad in scope and aimed at a maximal extent at public data. Social media provides such a data pool where public posts, engagements and inferences can be sourced to predict a response at the community level. This approach, already in use for marketing sentiment and response analysis (7) could be repurposed to predict, for instance, the number of cases of an outbreak, given a social media user’s inferred symptom.

If continuously monitored, social media mining could serve conjointly with metagenomic-based surveillance to identify any pathogens matching the outbreak inferred from social media data. We proceeded as such during a two-year metagenomic survey of Quebec City’s wastewater treatment influent. We compiled taxonomic profiles across weekly samples, both, respectively, indicating a surge in abundance for *Salmonella enterica* and a matching increase in predicted numbers of people experiencing salmonellosis symptoms.

A maximum abundance peak (first detected September 13^th^, 2023) was ultimately found among a subset of 17 samples collected between from September 2023 to January 2024 for whom AI-driven community analytics data was available and matching *Salmonella enterica* abundance fluctuations. Furthermore, we could reconstruct a strain-level assembly by re-assembling reads assigned to *Salmonella enterica* during the Sep 13th 2023 abundance peak. The pre-assembly read selection ultimately increased genome completeness and reduced contamination while also improving taxonomic assignment compared to a simple *de novo* binned assembly without sub-setting reads. We were also able to match its MLST profile to the one of *S. enterica* serovar *Typhimurium*, further confirming the presence of virulent non-typhoidal *Salmonella* in this abundance peak.

## Methods

### AI-driven community analytics

**Figure 1:**
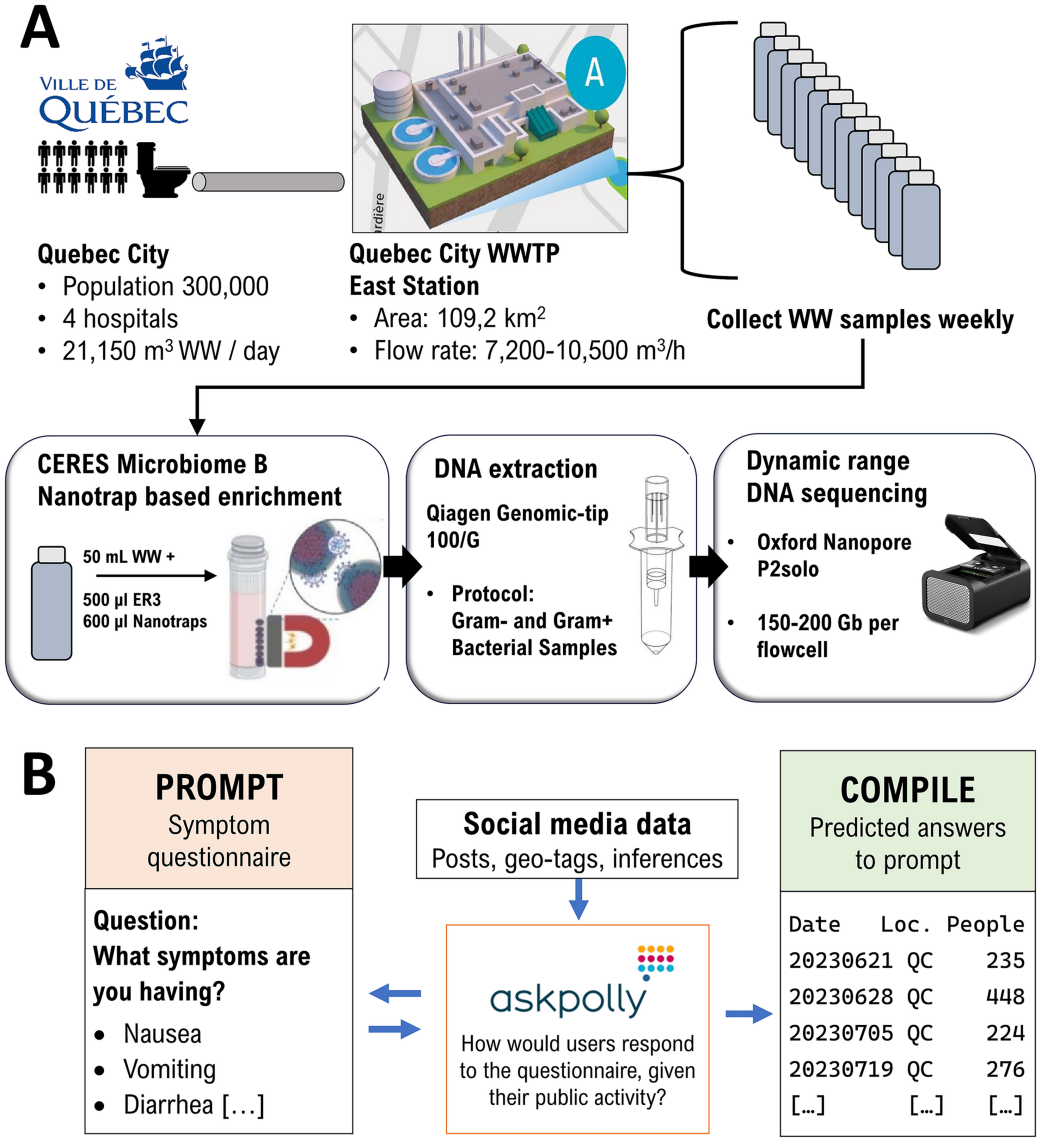
Joint workflow for continuous wastewater metagenome nanopore sequencing **(A)** and community-based AI-driven inferences **(B)**. WW, Raw wastewater; WWTP, Wastewater treatment plant; P2solo, Oxford Nanopore PromethION 2 solo portable DNA sequences.

AskPolly (8) is an AI driven, public opinion research platform, that was used to gather community analytics from X (formerly Twitter), Reddit, Tik Tok, BlueSky, and Mastodon (including Facebook Threads). A simplified schematic flowchart is included in Figure 1. AskPolly starts with an independent panel (9) across Canada. Posts from the individuals in the panel are collected and stored in a lexical and vector data lake. Data retrieval is based on a community level questionnaire, using the format of yes/no questions and possible symptoms a person could be experiencing (see Supplementary material for the full questionnaire). Exclusionary symptoms were also included to distinguish salmonellosis from other foodborne infections with a similar presentation such as botulism, hepatitis, listeriosis and staphylococcosis.

This questionnaire was repeated in French and English. All possible posts that could match the questionnaire were retrieved. Stances [32] were assigned by AskPolly to each post as Experiencing or Not Experiencing. Only posts classified as Experiencing were kept. The data was subset to Quebec City using a combination of self-disclosed information, geo-tags and inferences [33]. The output of this workflow is a count of people in Quebec City predicted as “Experiencing” non-typhoidal salmonellosis (Figure 1). AskPolly does not report counts below 100 at any given time to guarantee the anonymity of “Experiencing” social media users.

### Wastewater sampling procedure

From September 2023 to January 2024, weekly samples of 63 mL raw sewage water (SW) were collected from the Quebec City Wastewater Treatment East Station, which covers a population of 300,000 including 4 hospitals (Figure 1). Samples were taken directly from the WWTP’s influent intake pool with 100 mL Nalgene bottles strapped on long-handled tongs. Closed bottles were disinfected with Oxivir TB disinfectant spray, then stored in an isothermal cooler box to be transported to the laboratory within 1 h post-sampling. Immediately upon arrival, samples were transferred to a biocontainment level 2 cabinet, where total bacterial biomass was enriched by treatment with 600 μL NanoTrap Microbiome B magnetic beads per 50 mL SW (Ceres Nanosciences, Inc.).

### DNA extraction and quantification

Genomic DNA was extracted from cell pellets using the QIAGEN Genomic Tip 100/G kit with the manufacturer’s recommended protocol for Gram-negative and Gram-positive bacteria. DNA extracts were then size selected with the PacBio Short Read Eliminator XS (SREXS) magnetic beads, which completely excludes fragments below 5 kb to favor sequencing longer fragments. Size distribution was assessed before and after SREXS treatments with the FemtoPulse automated PFGE platform (Agilent Technologies). Final processed extracts were quantified with the Qubit dsDNA BR method (Thermo Fisher).

### Oxford nanopore long read sequencing

Throughout the monitoring timeline, samples were sequenced using Oxford Nanopore Technologies (ONT)‘s PromethION 2 solo platform. Briefly, 1 μg input DNA was processed with the Ligation Sequencing Kit v14 (SQK-LSK114) as recommended by the manufacturer’s protocol with a few minor adjustments (see Supplementary material). Prepared libraries were then sequenced on FLO-PRO002 (P2solo) and basecalled in real-time with MinKNOW v22.04, ONT’s proprietary firmware for sequencing. Base calling was performed using a minimum read length of 300 nt and a minimum Phred score of Q9.

### Taxonomic assignment and baseline monitoring

Reads from the SENTINEL time points were classified with Kraken2 v2.1.2 (10), using the pre-built NCBI Nucleotide k-mer index (761 GB, version 2023-05-22) built by Langmead et al. (https://benlangmead.github.io/aws-indexes/k2). Kraken2 reports were concatenated with the addition of a sample name column for further analysis with R (11) and RStudio (12).

By default, Kraken2 reports percent abundances by the total number of reads per time point. By doing so, reads that could not be classified due to poor quality or length remain included in the calculation. To circumvent this issue, which is more prevalent with Nanopore sequencing given its higher error rate relative to short read technologies (13), percent abundances were recalculated over reads that could be classified minimally to the domain level. We postulate that reads of sufficient quality should at least be recognizable as belonging to the known tree of life.

In addition, Kraken2 does not consider abundance cutoffs to report taxa, meaning that one spurious taxon from one single read could be reported. To avoid considering false positives, every count below 100 reads was discarded. As a further sanity check, the abundances should fluctuate over the time series, i.e., taxa should not appear and disappear completely. Therefore, taxa that were reported in less than 75% of the data points were discarded.

### *De novo* metagenome assembly

Nanopore reads from the SW2023-09-13 sample were quality filtered with chopper v0.7.0^1^ with minimum Phred score of 12 and minimum read length of 4,000 bp. Then, sequencing adapters were trimmed with PoreChop-ABI v0.5.0 (14). Pre-processed reads were then assembled *de novo* with Flye v2.9.5 (15) with the “—meta” flag to account for uneven contig coverage given the metagenomic nature of the sample. Finally, the draft assembly was polished with Medaka v2.0.1^2^ using pre-processed reads as input bases.

### Reference-guided genome reconstruction

If a known pathogen is present at sufficiently high levels in a SW sample, then it should be possible to reconstruct its genome sequence, or an approximate consensus of it, using a reference-guided assembly. We therefore pre-selected reads that were identified by Kraken2 as belonging to the same species. Then, only those reads were used for assembly. Briefly, seqtk^3^ was used to subset reads, in conjunction with grep over the Kraken2 reports to extract the list of read IDs. Then, those reads were aligned to a reference using minimap v2.28 (16) using profile “map-ont.” RefSeq genome accession GCF_000006945.2 was used for nontyphoid *Salmonella enterica, respectively.* After alignment, a draft consensus sequence was generated with racon v1.5.0 (17). A final polishing step was done with medaka v1.11.3 (see text footnote 2, respectively) to correct errors intrinsic to Nanopore sequencing. The resulting sequences were evaluated with CheckM v1.2.2 (18) to assess genome completeness in comparison with the reference used. Genome maps were produced with the ProkSee web annotation server (19). Classical MLST analysis was performed on reconstructed genomes along with the reference genome with PyMLST v2.1.6 (20) using the “claMLST command” and the PubMLST dataset for *Salmonella enterica*.^4^

## Results and discussion

### Taxonomic assignment and baseline monitoring

**Figure 2:**
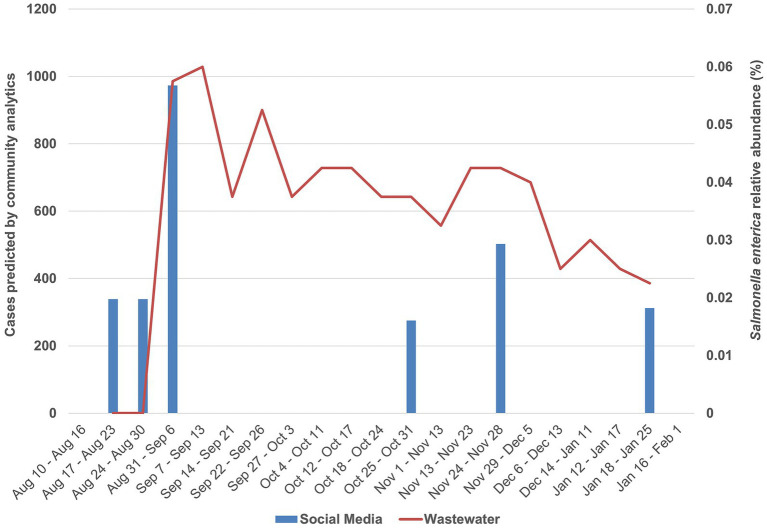
AI-driven community analytics for *Salmonella* symptoms overlap with metagenomic tracking of *Salmonella enterica*. Blue bars indicate community analytics with number of people reporting symptoms on the left axis. Missing blue bars are points below 100, which were considered too few to guarantee “Experiencing” social media users’ anonymity and were not reported by AskPolly. These can be interpreted as “less than 100”. The red line indicates wastewater pathogen measurement (expressed as percentage of all sample read counts), with relative abundance on the right axis.

From September 2023 to January 2024, 2,796 species were assigned to metagenomic reads throughout the time series (Supplementary Table 1). From those, *S. enterica* accounted for 0.03–0.04% of all classified reads (95% confidence interval, *N* = 18), making it 245th among detected species. Interestingly, this species reached a maximum abundance peak on September 13th, 2023, significantly above the upper confidence limit (0.05% respectively), indicating a surge in bacterial load above baseline fluctuations (Supplementary Figure 1). This peak, at the summer–fall transition, coincides with a surge in predicted counts of people experiencing symptoms reported by AskPolly (Figure 2), while also being in agreement with previous studies that reported a higher incidence of *Salmonella* cases in the food production chain during this period (21).

Of special interest, concerning *Salmonella*, the maximum abundance peak also coincides with two public health notices emitted by Canada’s health regulatory agency, which, respectively, reported surges of salmonellosis linked to cantaloupes, raw pet food and cattle in six provinces, including Quebec where this study took place (22, 23). Both notices were first emitted in October 2023, 1 month after the detected abundance peak. This indicates not only the concordance with clinical/epidemiological observations but also reinforces metagenomic monitoring and AI-driven community analytics as an early indicator of potential outbreaks.

The intrinsic design of ONT sequencing technology makes it more vulnerable to nucleotide mismatches and homopolymer indels, which may influence Kraken2’s ability to classify reads, as it does rely on exact k-mer matching. Nevertheless, the use of R10.4.1 flow cells with SQK-LSK114 chemistry helps bring reads to a median Phred score of Q15 (~3% per-nucleotide error rate) instead of the Q9-10 (~10–15% error rate) previously obtained with R9.4.1 flow cells with SQK-LSK109 chemistry. The longer reads (300 bp to several kbp) also help increase the number of k-mer matches with respect to 150–300 bp Illumina reads.

### AI-driven community analytics

Community data was collected from *Salmonella* symptoms across Canada from August 2023 through January 2024. The *Salmonella* questionnaire was run in both English and French. People reporting having symptoms in Quebec City are reported compared to the wastewater data from the same period (Figure 2). Community-reported symptoms consistently led the wastewater peaks by a week, suggesting that community data is a leading indicator of outbreaks, maybe even more so than metagenomic-based monitoring.

Nonetheless, the output of the symptom questionnaire is by any means insufficient *per se* for appropriate identification of a pathogen causing an outbreak, which is why we consider these approaches as complementary: AI to detect potential outbreaks; metagenomics to identify potential causative agents. It can also put public health stakeholders on alert by triggering them to increase their monitoring effort. The advantage of both social media data and wastewater metagenomic data is that both can be monitored and archived continuously, with further analysis in the event of an outbreak.

### Whole metagenome shotgun assembly

Read-based metagenomic surveillance helped track *S. enterica* at the species level. However, non-virulent conspecific strains could also contribute to the abundance peak, given the variable accuracy of read classifiers at the species level (24). Kraken2 is particularly affected by Nanopore sequence error rates as its algorithm performs exact k-mer matches to classify sequences (10). Therefore, we attempted to *de novo* assemble the whole metagenome shotgun sequence for the 2023-09-13 sample to further increase taxonomic resolution. Given enough sequencing depth, metagenome-assembled genomes can be reconstructed (25, 26). For instance, MaxBin and MetaBAT2 regroup contigs having similar G-C content, depth of coverage, and tetranucleotide usage statistics, assuming that contigs should share these properties if belonging to the same source organism (25, 26). However, difficulties arise when co-occurring organisms share these properties, or when the organism of interest is in low abundance among a large set of reads (27).

As expected, this assembly did not recover any contigs classified as *S. enterica*, even though Flye was run with the –meta flag to allow for uneven coverage across contigs (15). This could be attributable to the very low abundance of those enteric pathogens within the sample (less than 0.5% of all reads). Indeed, reads from the SW2023-09-13 sample revealed that it was largely populated by bacteria (75% of all read counts), among which Pseudomonadota, the phylum comprising *S. enterica*., accounted for 43% of all bacterial reads (Supplementary Figure 2). The Enterobacteriaceae family represented 5% of all Pseudomonadota within this sample (Supplementary Figure 2). *S. enterica* reads, regardless of strains, accounted for 1%, respectively, of all Enterobacteriaceae reads (Supplementary Figure 2), thereby illustrating a case where most of the dataset does not represent the genomic target of interest.

We then proceeded to assemble only reads that were assigned as *S. enterica* using the same *de novo* assembly approach. This approach seemed viable as there were 10,574 reads (87,590,049 bp) assigned to *S. enterica.* This subassembly would have had an expected coverage of 17.5x coverage assuming 5 Mb genome size. However, this subassembly was suboptimal for both species. The *S. enterica* subassembly yielded 166 contigs totalling 3,72 Mbp with 9x coverage (less than the expected genome size of 5 Mb for *S. enterica*). Given that SW2023-09-13 was the time point linked with an abundance peak for the whole time series, we do not expect *de novo* subassemblies to perform better on other samples. In addition, human (host) reads accounted for 1.56% of total counts in metagenomic reads from the 2023-09-13 abundance peak (6th most abundant). This indicates that the CERES Microbiome B Nanotrap-based microbial enrichment retained some non-target biomass. A possible solution would be to use Oxford Nanopore sequencing in adaptive sampling mode, where an input genome sequence can be provided to reject host DNA reads in real time (and therefore enrich microbial metagenomic reads further).

Instead of using *de novo* assembly, we attempted to reconstruct genome sequences by reference-guided assembly using the genome of type strain *S. enterica* subsp. *enterica* sv. Typhimurium LT2 and reads from the 2023-09-13 abundance peak as input.

### Reference-guided genome reconstruction

Given the large size of this read set (125 gigabases) and that *S. enterica* reads account for 0.04% of all reads (Supplementary Table 2), we obtained an incomplete assembly with abnormal feature counts with respect to their reference (Supplementary Table 3). However, when only considering reads assigned to these species, we obtained 6.5X coverage, enough to re-attempt a reference-based assembly. This assembly was 95% complete (Supplementary Table 3) and agreed with the main characteristics of their respective reference (Supplementary Figure 3).

Furthermore, classical MLST was performed to assess the possibility of typing reference-based assemblies (Supplementary Table 4). In both cases, when all reads from the sample are used to map against the reference, 0/7 MLST loci were identified. In the assembly with *S. enterica* reads only, 5 loci out of 7 match the profile of type strain *S. enterica* subsp. *enterica* strain LT2 (serovar Typhimurium), suggesting high relatedness to other enteropathogenic non-typhoidal strains. We do agree, however, that the degree of representativeness and chimerism in genome reconstruction is a key challenge in metagenomics (28) that can impact the meaningfulness of strain typing on MAGs.

Nevertheless, the final reference-guided assembly had 0.1% contamination and 0% strain heterogeneity (Supplementary Table 3), suggesting this assembly is not a chimeric consensus of various strains. However, closer investigation into the classical MLST profile of *S. enterica* SW2023-09-13 MAG revealed 2 of 7 alleles identified were unidentifiable. Those could indeed be a consensus of reads from multiple alleles beyond recognition by the used MLST scheme or the result of mis-assembly in those loci. There does exist software for deconvoluting multiallelic profiles (i.e., haplotype phasing), though these are usually designed for polyploid eukaryotic genomes (29). A similar approach, which would phase the resulting consensus assembly, could further allow strain-level inference.

### Limitations and perspectives

Even though AI-driven social media analytics and whole metagenome monitoring could be combined as part of a pathogen surveillance workflow, several limitations remained to be addressed for such a program to be deployed at the nationwide level. For instance, this workflow relies heavily on social media data, whereas social media users are not a random sample of the population (30). Even though 90% of Canadians are social media users (31), there is substantial variation in usage by age, gender, socio-economic status and geographical location (8).

Furthermore, social media users do not typically report nominal healthy states, which could lead to some form of survivorship bias in the data used by AskPolly. This is not unlike longitudinal mental health surveys during the COVID-19 pandemic, where people affected by mental health conditions were more likely to self-report in such studies (9). Even though the AskPolly prompt questionnaires include exclusionary symptoms, the capacity to differentiate salmonellosis from other similar questions remains uncertain, as the AskPolly-assigned stances (Experiencing vs. Not experiencing) ultimately depend on how social media users accurately report their symptoms without formal medical knowledge. Finally, the “below 100” anonymity threshold, while preventing user traceback, creates discontinuities in time series that may limit the assessment of common trends between social media analytics and metagenomic monitoring.

## Conclusion

In this study, AI-driven community analytics and high-throughput long-read metagenomic surveillance of Quebec City’s wastewater microbiome both indicated similar fluctuations of predicted salmonellosis cases and *Salmonella enterica* relative abundance in wastewater. Moreover, both approaches detected a maximum on September 13th, 2023, 1 month before a nationwide *Salmonella* food recall by Canadian health authorities. Binned-read reference-based assembly further confirmed the presence of non-typhoidal *Salmonella enterica* in the wastewater influent during this period. Moreover, community indicators led the metagenomic peaks by a week. We believe that AI-driven community analytics and wastewater metagenomics monitoring could become part of a nationwide surveillance pipeline for health authorities in the short-medium term. Though promising, several technical and methodogical improvements remain to be achieved to improve both the reliability of AI-driven community analytics and characterization of the causative pathogen to the strain level *via* metagenomic tracking and genomic reconstruction.

## Data Availability

The datasets presented in this study can be found in online repositories. The names of the repository/repositories and accession number(s) can be found in the article/Supplementary material.

## Footnotes

1
https://github.com/wdecoster/chopper

2
https://github.com/nanoporetech/medaka

3
https://github.com/lh3/seqtk

4
https://pubmlst.org/data/

## References

[1] Li T Qiang N Bao Y Li Y Zhao S Chong KC . Global burden of enteric infections related foodborne diseases, 1990–2021: findings from the global burden of disease study 2021. Sci One Health. (2024) 3:100075. doi: 10.1016/j.soh.2024.100075, PMID: 39282625 PMC11402448

[2] Buzby JC Roberts T. The economics of enteric infections: human foodborne disease costs. Gastroenterology. (2009) 136:1851–62. doi: 10.1053/j.gastro.2009.01.074, PMID: 19457414

[3] Lamichhane B Mawad AMM Saleh M Kelley WG Harrington PJ Lovestad CW . Salmonellosis: an overview of epidemiology, pathogenesis, and innovative approaches to mitigate the antimicrobial resistant infections. Antibiotics. (2024) 13:76. doi: 10.3390/antibiotics13010076, PMID: 38247636 PMC10812683

[4] Joseph TA Pe’er I. An introduction to whole-metagenome shotgun sequencing studies. Methods Mol Biol. (2021) 2243:107–22. doi: 10.1007/978-1-0716-1103-6_6, PMID: 33606255

[5] Tornimbene B Leiva Rioja ZB Brownstein J Dunn A Faye S Kong J . Harnessing the power of artificial intelligence for disease-surveillance purposes. BMC Proc. (2025) 19:7. doi: 10.1186/s12919-025-00320-w, PMID: 40050981 PMC11887143

[6] Gundogmusler A Bayindiroglu F Karakucukoglu M. Mathematical foundations of hallucination in transformer-based large language models for improvisation. TechRxiv. (2024) 2024:486. doi: 10.36227/techrxiv.171925719.97800486/v1

[7] Zachlod C Samuel O Ochsner A Werthmüller S. Analytics of social media data – state of characteristics and application. J Bus Res. (2022) 144:1064–76. doi: 10.1016/j.jbusres.2022.02.016

[8] Schimmele C Fonberg J Schellenberg G. Canadians’ assessments of social media in their lives. Ottawa: Statistics Canada.

[9] Czeisler MÉ Wiley JF Czeisler CA Rajaratnam SMW Howard ME. Uncovering survivorship bias in longitudinal mental health surveys during the COVID-19 pandemic. Epidemiol Psychiatr Sci. (2021) 30:e45. doi: 10.1017/S204579602100038X, PMID: 34036933 PMC8207539

[10] Wood DE Lu J Langmead B. Improved metagenomic analysis with kraken 2. Genome Biol. (2019) 20:257. doi: 10.1186/s13059-019-1891-0, PMID: 31779668 PMC6883579

[11] R Core Team. R: A language and environment for statistical computing. Vienna, Austria: R Foundation for Statistical Computing (2019).

[12] RStudio Team. RStudio: Integrated development environment for R. Boston, MA: RStudio, Inc. (2016).

[13] Zhang T Li H Jiang M Hou H Gao Y Li Y . Nanopore sequencing: flourishing in its teenage years. J Genet Genomics. (2024) 51:1361–74. doi: 10.1016/j.jgg.2024.09.007, PMID: 39293510

[14] Bonenfant Q Noé L Touzet H. Porechop_ABI: discovering unknown adapters in Oxford nanopore technology sequencing reads for downstream trimming. Bioinform Adv. (2023) 3:vbac085. doi: 10.1093/bioadv/vbac085, PMID: 36698762 PMC9869717

[15] Kolmogorov M Yuan J Lin Y Pevzner PA. Assembly of long, error-prone reads using repeat graphs. Nat Biotechnol. (2019) 37:540–6. doi: 10.1038/s41587-019-0072-8, PMID: 30936562

[16] Li H. Minimap2: pairwise alignment for nucleotide sequences. Bioinformatics. (2018) 34:3094–100. doi: 10.1093/bioinformatics/bty191, PMID: 29750242 PMC6137996

[17] Vaser R Sović I Nagarajan N Šikić M. Fast and accurate de novo genome assembly from long uncorrected reads. Genome Res. (2017) 27:737–46. doi: 10.1101/gr.214270.116, PMID: 28100585 PMC5411768

[18] Parks DH Imelfort M Skennerton CT Hugenholtz P Tyson GW. CheckM: assessing the quality of microbial genomes recovered from isolates, single cells, and metagenomes. Genome Res. (2015) 25:1043–55. doi: 10.1101/gr.186072.114, PMID: 25977477 PMC4484387

[19] Grant JR Enns E Marinier E Mandal A Herman EK Chen CY . Proksee: in-depth characterization and visualization of bacterial genomes. Nucleic Acids Res. (2023) 51:W484–92. doi: 10.1093/nar/gkad326, PMID: 37140037 PMC10320063

[20] Biguenet A Bordy A Atchon A Hocquet D Valot B. Introduction and benchmarking of pyMLST: open-source software for assessing bacterial clonality using core genome MLST. Microb Genom. (2023) 9:001126. doi: 10.1099/mgen.0.001126, PMID: 37966168 PMC10711306

[21] Flores Monter YM Chaves A Arellano-Reynoso B López-Pérez AM Suzán-Azpiri H Suzán G. Edaphoclimatic seasonal trends and variations of the Salmonella spp. infection in northwestern Mexico. Infect Dis Model. (2021) 6:805–19. doi: 10.1016/j.idm.2021.05.002, PMID: 34258482 PMC8237282

[22] Public Health Agency of Canada. *Public health notice: outbreak of Salmonella infections linked to Malichita and Rudy brand cantaloupes*. (2023). Available online at: https://www.canada.ca/en/public-health/services/public-health-notices/2023/outbreak-salmonella-infections-malichita-cantaloupes.html (Accessed July 9, 2024).

[23] Public Health Agency of Canada. *Public health notice: outbreak of extensively drug-resistant Salmonella infections linked to raw pet food and contact with cattle*. (2024). Available online at: https://www.canada.ca/en/public-health/services/public-health-notices/2023/outbreak-salmonella-infections-under-investigation.html (Accessed July 9, 2024).

[24] Van Uffelen A Posadas A Roosens NHC Marchal K De Keersmaecker SCJ Vanneste K. Benchmarking bacterial taxonomic classification using nanopore metagenomics data of several mock communities. Sci Data. (2024) 11:864. doi: 10.1038/s41597-024-03672-8, PMID: 39127718 PMC11316826

[25] Wu Y-W Simmons BA Singer SW. MaxBin 2.0: an automated binning algorithm to recover genomes from multiple metagenomic datasets. Bioinformatics. (2016) 32:605–7. doi: 10.1093/bioinformatics/btv638, PMID: 26515820

[26] Kang DD Li F Kirton E Thomas A Egan R An H . MetaBAT 2: an adaptive binning algorithm for robust and efficient genome reconstruction from metagenome assemblies. PeerJ. (2019) 7:e7359. doi: 10.7717/peerj.7359, PMID: 31388474 PMC6662567

[27] Vosloo S Huo L Anderson CL Dai Z Sevillano M Pinto A. Evaluating de novo assembly and binning strategies for time series drinking water metagenomes. Microbiol Spectr. 9:e01434–21. doi: 10.1128/Spectrum.01434-21, PMID: 34730411 PMC8567270

[28] Chang T Gavelis GS Brown JM Stepanauskas R. Genomic representativeness and chimerism in large collections of SAGs and MAGs of marine prokaryoplankton. Microbiome. (2024) 12:126. doi: 10.1186/s40168-024-01848-3, PMID: 39010229 PMC11247762

[29] Vences M Patmanidis S Schmidt J-C Matschiner M Miralles A Renner SS. Hapsolutely: a user-friendly tool integrating haplotype phasing, network construction, and haploweb calculation. Bioinformatics Adv. (2024) 4:vbae083. doi: 10.1093/bioadv/vbae083, PMID: 38895561 PMC11184345

[30] Mai P. *The state of social Media in Canada 2022*. *Social Media Lab*. (2022). Available online at: https://socialmedialab.ca/2022/09/14/survey-finds-canadians-are-spending-less-time-on-social-media-but-tiktok-is-the-exception/ (Accessed October 22, 2025).

[31] Denham B. *Trends: social Media in Canada*. Environics Research. (2024). Available online at: https://environics.ca/insights/articles/2024-trends-social-media-in-canada/ (Accessed October 22, 2025).

