# Supplementary material for: Leveraging artificial intelligence community analytics and nanopore metagenomic surveillance to monitor early enteropathogen outbreaks
Source: Front Public Health. 2025 Nov 26;13:1675080. doi: 10.3389/fpubh.2025.1675080 (PMC12689962; doi:10.3389/fpubh.2025.1675080)
Supplement: Supplementary file 1 [file Data_Sheet_1.docx]

*Leveraging AI community analytics and Nanopore metagenomic surveillance to monitor early enteropathogen outbreaks*

**Supplementary Information**

**Jeff Gauthier^1,*^, Sima Mohammadi^1^, Irena Kukavica-Ibrulj^1^, Brian Boyle^1^,
Chrystal Landgraff^2^, Lawrence Goodridge^3^, Kenton White^4^, Benjamin Chapman^4^,
Roger C. Levesque^1,*^**

1. Département de microbiologie-infectiologie et d'immunologie, Institut de Biologie Intégrative et des Systèmes, Université Laval, Quebec, Canada
2. National Microbiology Laboratory, Public Health Agency of Canada, Winnipeg, MB, Canada
3. Food Science Department, University of Guelph, Guelph, ON, Canada.
4. Advanced Symbolics Inc., Ottawa, ON, Canada

* Corresponding authors: [rclevesq@ibis.ulaval.ca](mailto:rclevesq@ibis.ulaval.ca) ; [jeff.gauthier.1@ulaval.ca](mailto:jeff.gauthier.1@ulaval.ca)

# Supplementary Methods

## AskPolly full prompt questionnaire for nontyphoidal salmonellosis:

1. *Symptoms*
   1. *Have you experienced diarrhea, abdominal cramps, or fever?*
   2. *Did the diarrhea have blood in it?*
   3. *Have you had nausea, vomiting, or chills?*
2. *Exposure*
   1. *Have you consumed raw or undercooked eggs, poultry, or meat in the past 10 days?*
   2. *Have you eaten any unwashed fruits or vegetables?*
   3. *Did you drink unpasteurized milk or juice?*
   4. *Have you been in contact with reptiles, amphibians, or pet birds?*
   5. *Have you traveled internationally recently?*
3. *Incubation Period*
   1. *When did symptoms begin in relation to the last food you ate (typically 6–48 hours)?*
4. *Exclusionary Symptoms:*
   1. *Are you experiencing numbness, tingling, or muscle weakness (e.g., botulism)?*
   2. *Have you had jaundice or dark urine (e.g., hepatitis)?*
   3. *Do you have neurological symptoms such as blurred vision or difficulty swallowing?*
   4. *Are you experiencing sudden vomiting without diarrhea (e.g., Staphylococcus aureus)?*

# Supplementary Figures

##
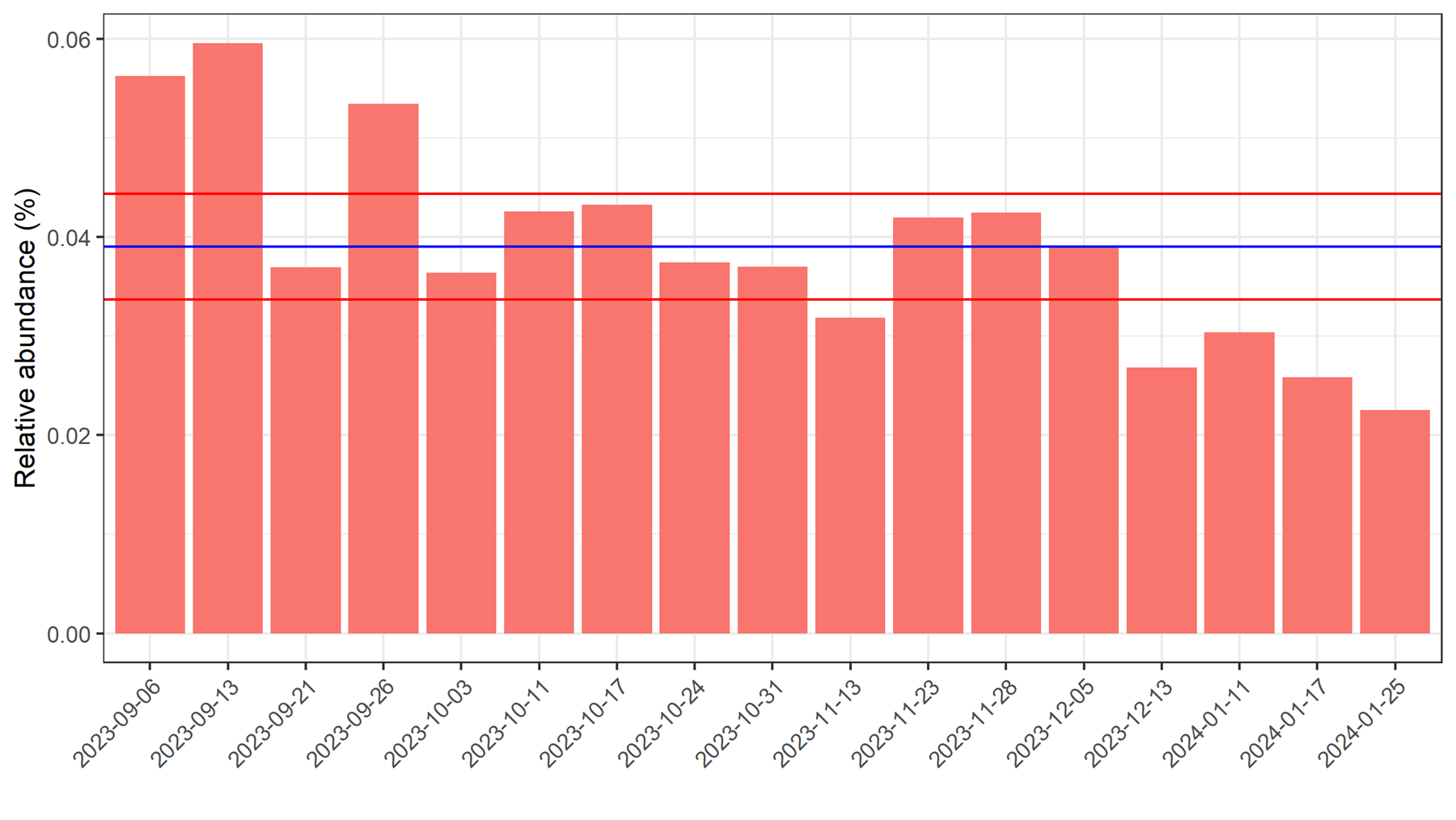
**Suppl. Figure 1. Weekly monitoring of *Salmonella enterica* reads in Quebec City raw sewage water samples from September 2023 to January 2024.** Blue line: average relative abundance, here defined as the percentage of classified reads within a sample. Red lines: 95% confidence interval.


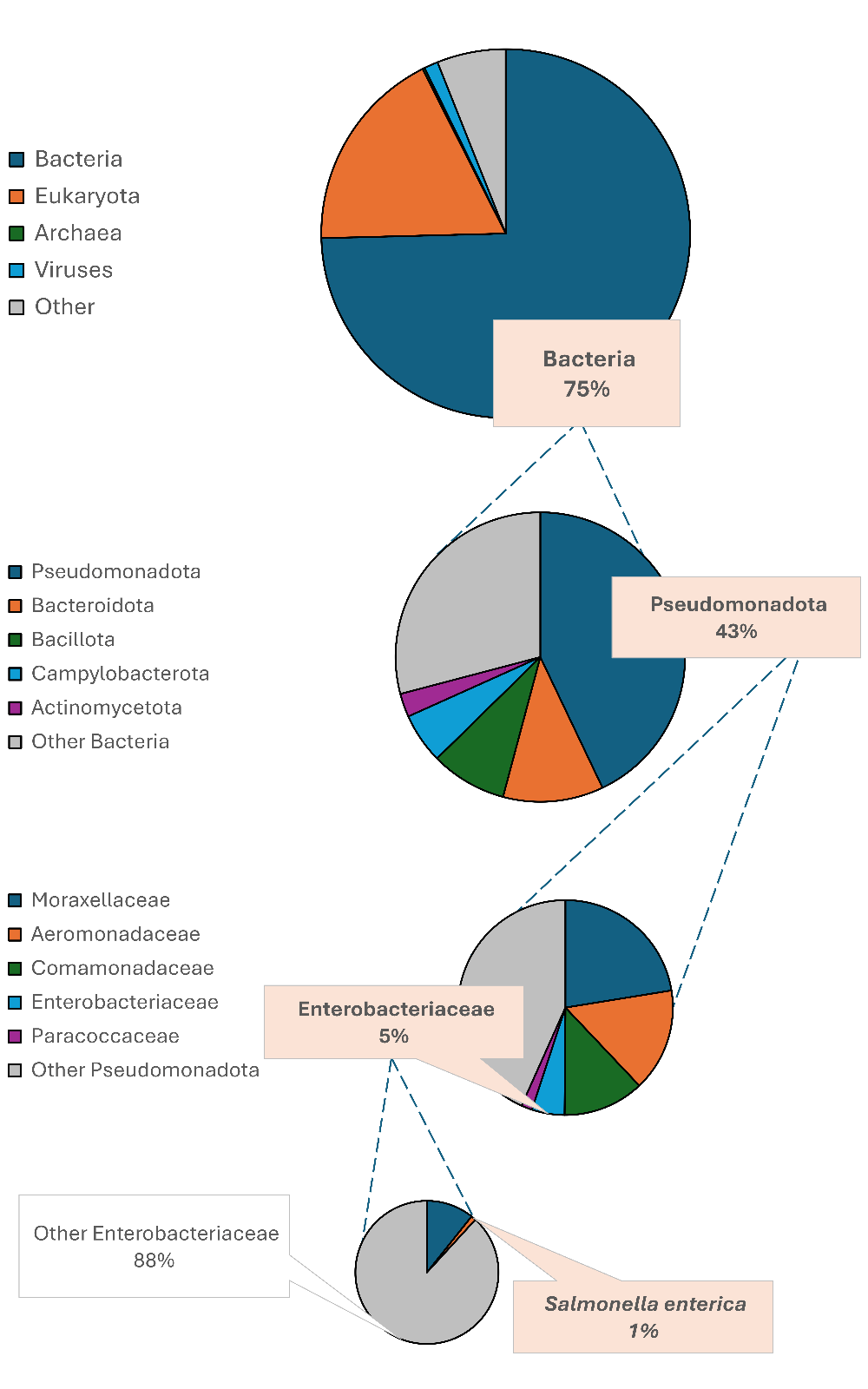


## **Suppl. Figure 2. *Salmonella enterica* reads from sample SW2023-09-13, expressed as percent read counts within each taxonomic rank.** Both species account for 12% of reads classified as Enterobacteriaceae, themselves representing 5% of all read counts within the phylum Pseudomonadota. This phylum itself accounts for 43% of all read counts assigned to Bacteria, themselves comprising 75% of all reads counts within this sample.

##
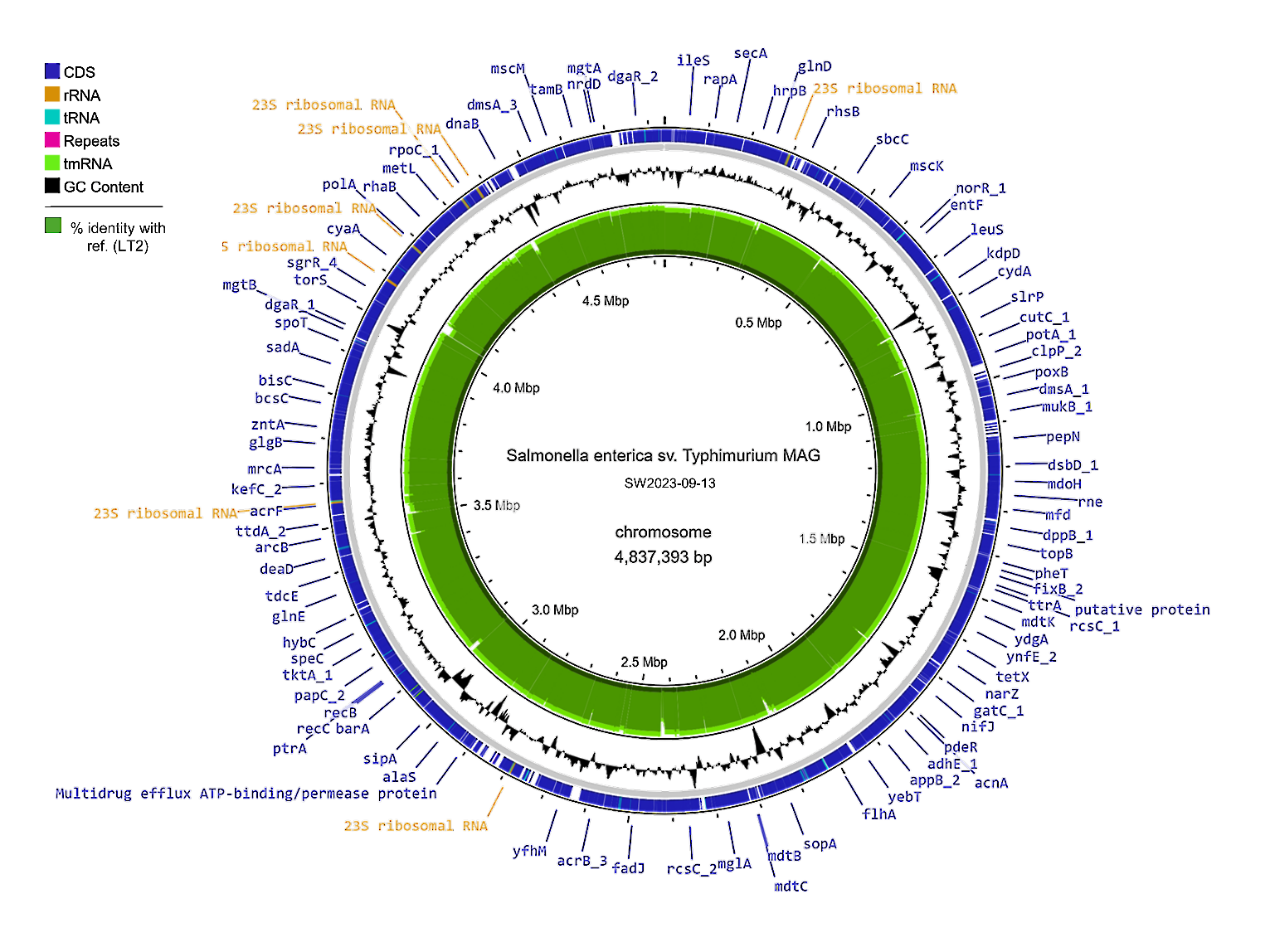
**Suppl. Figure 3.** **Map of *Salmonella enterica* MAG SW20230913.** Outer ring: reconstructed genome sequence. Inner ring: reference GCF_000006945.2 for nontyphoid *Salmonella enterica*).

# Supplementary Tables

## **Suppl. Table 1.** Overall species abundance, estimated with Kraken2 by percent read counts, throughout Quebec City wastewater samples sequenced from September 2023 to January 2024.

| Species assignments | Rank | N samples present | % of read counts | 95% confidence interval |
| --- | --- | --- | --- | --- |
| *Moraxella osloensis* | 1 | 18 | 7.12 | 2.02 |
| *Acinetobacter johnsonii* | 2 | 18 | 4.43 | 0.46 |
| *Arcobacter suis* | 3 | 18 | 2.97 | 0.91 |
| *Aeromonas media* | 4 | 18 | 2.03 | 0.29 |
| *Aliarcobacter cryaerophilus* | 5 | 18 | 1.96 | 0.35 |
| *Homo sapiens* | 6 | 18 | 1.56 | 0.62 |
| *Aeromonas caviae* | 7 | 18 | 1.50 | 0.30 |
| *Acinetobacter sp. TTH0-4* | 8 | 18 | 1.50 | 0.72 |
| *Lactococcus raffinolactis* | 9 | 18 | 1.49 | 0.56 |
| *Tolumonas auensis* | 10 | 18 | 1.44 | 0.36 |
| *…* | … | … | … | … |
| *Escherichia coli* | 32 | 18 | 0.40 | 0.06 |
| *Salmonella enterica* | 245 | 17 | 0.04 | 0.01 |
| *[…]* | […] | […] | […] | […] |
| *TOTAL* | 2,796 | - | - | - |

## **Suppl. Table 2.** Proportions of reads classified as *Salmonella enterica* in a predicted abundance peak common to both pathogens of interest (SW2023-09-13).

| **Reads** | **Total bases** | **% bases kept** | **Expected genome size** | **Estimated coverage** |
| --- | --- | --- | --- | --- |
| SW2023-09-13,  **all reads** | 124 502 695 920 bp | - | - | - |
| SW2023-09-13,  ***S. enterica* reads only** | 31 819 198 bp | **0,03 %** | 4 951 383 bp | **6,42x** |

## **Suppl. Table 3.** Assembly metrics and features throughout reference-guided genome reconstruction of *S. enterica subsp. enterica* using nanopore reads from the 2023-09-13 data set.

| **Assembly** | **Contigs** | **Bases** | **CDS** | **CRISPR** | **tRNA** | **Completeness**  **(CheckM)** | **Contamination**  **(CheckM)** | **Strain heterogeneity**  **(CheckM)** |
| --- | --- | --- | --- | --- | --- | --- | --- | --- |
| **Reference (*S. enterica* subsp. *enterica* strain LT2)** | **2** | **4 951 383 bp** | **4 627** | **0** | **86** | **100 %** | **0.04 %** | **0 %** |
| SW2023-09-13 (all reads mapped) | 2 | 4 535 144 bp | 8 942 | 0 | 78 | 67.2 % | 0.04 % | 0 % |
| **SW2023-09-13**  **(*S. enterica* reads only)** | **2** | **4 930 755 bp** | **5 130** | **0** | **79** | **95.1 %** | **0.10 %** | **0 %** |

## **Suppl. Table 4.** Multilocus sequence typing (MLST) analysis for reference-based assemblies with whole metagenome shotgun reads from wastewater sample SW2023-09-13.

|  |  |  |  |  |  |  |  |
| --- | --- | --- | --- | --- | --- | --- | --- |
| **Sample** | ***aroC*** | ***dnaN*** | ***hemD*** | ***hisD*** | ***purE*** | ***sucA*** | ***thrA*** |
| Reference (*S. enterica subsp. enterica* strain LT2) | 10 | 7 | 12 | 9 | new | 9 | 2 |
| SW2023-09-13 (all reads mapped) | NA | NA | NA | NA | NA | NA | NA |
| SW2023-09-13 (S. enterica reads only) | 10 | 7 | 12 | 9 | new | NA | NA |

MLST schemas were retrieved from the “Salmonella spp.” classical multilocus dataset from PubMLST (<https://pubmlst.org/data/>). NA cells indicate missing data, which may be attributable to sequencing error and/or genome incompleteness (see Tables 2 and 3). Loci that were present but not matching alleles in the dataset were automatically labeled as “new”.
